# Proteomic Characterization of Proliferation Inhibition of Well-Differentiated Laryngeal Squamous Cell Carcinoma Cells Under Below-Background Radiation in a Deep Underground Environment

**DOI:** 10.3389/fpubh.2020.584964

**Published:** 2020-10-30

**Authors:** Jifeng Liu, Tengfei Ma, Mingzhong Gao, Yilin Liu, Jun Liu, Shichao Wang, Yike Xie, Qiao Wen, Ling Wang, Juan Cheng, Shixi Liu, Jian Zou, Jiang Wu, Weimin Li, Heping Xie

**Affiliations:** ^1^Department of Otolaryngology Head and Neck Surgery, West China Hospital, Sichuan University, Chengdu, China; ^2^Deep Underground Space Medical Center, West China Hospital, Sichuan University, Chengdu, China; ^3^College of Water Resources and Hydropower, Sichuan University, Chengdu, China; ^4^Department of Ophthalmology, West China Hospital, Sichuan University, Chengdu, China; ^5^Institute of Deep Earth Science and Green Energy, Shenzhen University, Shenzhen, China

**Keywords:** deep-underground, below background radiation, proteomics, cell proliferation, stress, ribosome, FD-LSC-1 cell

## Abstract

**Background:** There has been a considerable concern about cancer induction in response to radiation exposure. However, only a limited number of studies have focused on the biological effects of below-background radiation (BBR) in deep underground environments. To improve our understanding of the effects of BBR on cancer, we studied its biological impact on well-differentiated laryngeal squamous cell carcinoma cells (FD-LSC-1) in a deep underground laboratory (DUGL).

**Methods:** The growth curve, morphological, and quantitative proteomic experiments were performed on FD-LSC-1 cells cultured in the DUGL and above-ground laboratory (AGL).

**Results:** The proliferation of FD-LSC-1 cells from the DUGL group was delayed compared to that of cells from the AGL group. Transmission electron microscopy scans of the cells from the DUGL group indicated the presence of hypertrophic endoplasmic reticulum (ER) and a higher number of ER. At a cutoff of absolute fold change ≥ 1.2 and *p* < 0.05, 807 differentially abundant proteins (DAPs; 536 upregulated proteins and 271 downregulated proteins in the cells cultured in the DUGL) were detected. KEGG pathway analysis of these DAPs revealed that seven pathways were enriched. These included ribosome (*p* < 0.0001), spliceosome (*p* = 0.0001), oxidative phosphorylation (*p* = 0.0001), protein export (*p* = 0.0001), thermogenesis (*p* = 0.0003), protein processing in the endoplasmic reticulum (*p* = 0.0108), and non-alcoholic fatty liver disease (*p* = 0.0421).

**Conclusion:** The BBR environment inhibited the proliferation of FD-LSC-1 cells. Additionally, it induced changes in protein expression associated with the ribosome, gene spliceosome, RNA transport, and energy metabolism among others. The changes in protein expression might form the molecular basis for proliferation inhibition and enhanced survivability of cells adapting to BBR exposure in a deep underground environment. RPL26, RPS27, ZMAT2, PRPF40A, SNRPD2, SLU7, SRSF5, SRSF3, SNRPF, WFS1, STT3B, CANX, ERP29, HSPA5, COX6B1, UQCRH, and ATP6V1G1 were the core proteins associated with the BBR stress response in cells.

## Introduction

Humans are exposed to radiation that originates from natural and man-made sources (1). There has been a considerable concern about cancer induction in response to radiation exposure, which is one of the potential harmful effects of radiation (2). A linear no-threshold (LNT) relationship, which is an assumption model, was formed between dose and risk values to estimate the risk of cancer induced by low doses of radiation (1). However, increasing evidence suggests that the risks of low doses of radiation might not strictly conform to an LNT (2). In particular, the relevant biological research data has been collected from experiments conducted in deep underground laboratories (DUGLs), which were originally used to conduct particle, astroparticle, or nuclear physics experiments that require an environment with significantly low interference by cosmic ray particles (3). Biological experiments conducted in DUGLs revealed the detrimental effects of an environment with low background radiation on cultures (3). However, the limited number of studies that focus on this issue has made it difficult to draw a meaningful conclusion about the biological effects of below-background radiation (BBR).

To better serve the national interests of China related to the exploration of deep underground spaces and resources, a deep underground medical laboratory was established at Erdaogou Mine, Jiapigou Minerals Limited Corporation (CJEM) of China National Gold Group Corporation in northeast China (3). Owing to the rocky cover of 1,470 m, the cosmic radiation exposure in the DUGL at CJEM was negligible (4). The terrestrial γ-ray dose rate radiation in the DUGL (0.04 μSv/h) was only one-third of that in the above-ground laboratory (AGL; 0.15 μSv/h), which was constructed as a control laboratory in an office building near the entrance of the CJEM facility (4). We determined the environmental characteristics and tested the feasibility of conducting experiments at the DUGL at CJEM in December 2017 (3). The other environmental parameters showed that relative humidity (DULG/AGL = 99%/57.2%) (*p* < 0.001), air pressure (DULG/AGL = 1118.2 hPa/951.9 hPa) (*p* < 0.001), and concentration of CO_2_ (DULG/AGL = 951.9 ppm/540.11 ppm] and radon gas (DULG/AGL = 4.0 pCi/L/1.25 pCi/L) (*p* < 0.001) were significantly higher in the DUGL compared to the AGL (4). However, O_2_ concentration in the DUGL (20.8%) and AGL (20.6%) was not significantly different (4).

Initial studies showed that Chinese hamster V79 cells could be successfully cultured in the DUGL and presented with proliferation inhibition (3). In addition, quantitative proteomic analyses revealed that the differentially abundant proteins (DAPs) in V79 cells from the DUGL and AGL groups were associated with the ribosome pathway, RNA transport, and oxidative phosphorylation (OXPPL) among others. The alteration in protein expression might form the basis for V79 cell growth delay and stress response under BBR in the deep underground environment. However, the cells studied in the DUGL were normal cells. Only a limited number of studies have investigated the responses in cancer cells under such environments, in which background radiation is shielded.

We selected well-differentiated laryngeal squamous cell carcinoma cells (FD-LSC-1), which are moderately sensitive to radiation (4), to observe the biological effect of BBR in the DUGL at the CJEM facility. The growth curve, morphological, and quantitative proteomic experiments were performed on FD-LSC-1 cells cultured in the DUGL and AGL. These data might provide novel insights into the relationship between radiation and cancers in a BBR environment.

## Materials and Methods

The methods followed for cell culture, growth curve, transmission electron microscopy (TEM), tandem mass tag (TMT) protein quantification, and parallel reaction monitoring (PRM) experiments have been presented in one of our previous studies (4).

### Cell Culture

**Figure 1:**
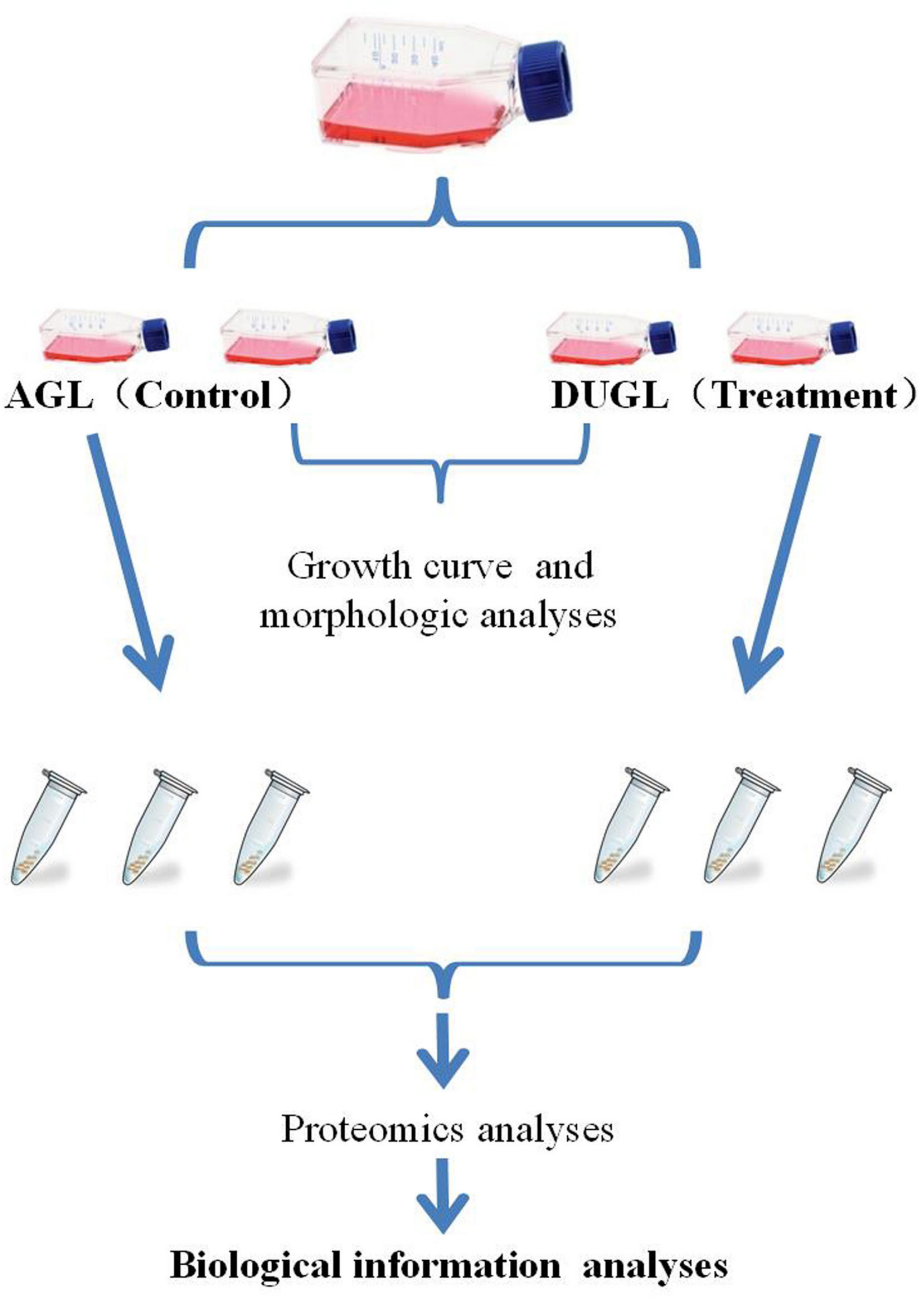
Flow chart of the study design. AGL, above-ground laboratory; DUGL, deep underground laboratory.

Briefly, FD-LSC-1 cells were purchased from the Shanghai branch of the Chinese Academy of Science. The frozen FD-LSC-1 cells were revived and cultured in Dulbecco's modified Eagle medium (Gibco, USA) supplemented with 10% fetal calf serum (Gemini, USA), 50 U dm^−3^ penicillin, and streptomycin (Gibco, USA). Once the cells reached above 80% confluence, they were passaged. Next, the cultures were divided among four bottles. Two bottles were assigned randomly for culturing in the DUGL or AGL. The bottles for DUGL were transferred from AGL to DUGL in an incubator, which took about 1.5–2 h from the AGL to DUGL. Then the cells were maintained in incubators at the two locations at 37°C and 5% CO_2._ The culture from one bottle from each laboratory was used to analyze the proliferation ability and morphological changes. The culture from the second bottle from each laboratory was used for passage when the cells were more than 80% confluent. After 4 days of culture in the DUGL or AGL, three bottles of the cell samples cultured in each location were collected on site for proteomic analyses. The flow chart for the study is presented in Figure 1.

### Growth Curve

Cell Counting Kit-8 (CCK-8) (MCE, USA) was used to compare the proliferative potential of FD-LSC-1 cells from the DUGL and AGL groups. Briefly, 200 μL of cell suspension at a concentration of 5 × 10^5^ cells/mL, was added to each well in a 96-well plate. The cells were cultured in the incubator. The CCK-8 reagent was added to the five replicate wells (10 μL/well) every day for the following 7 days; then, the plates were incubated for 4 h in the incubator at both the locations. The absorbance of each well was measured at 450 nm (OD_450_). On each day and at each location, the absorbance of cultures in the five replicate wells was measured to analyze the proliferation potential of the cells. The average value of five replicate absorbance values recorded each day was used to construct the growth curve.

### TEM

The FD-LSC-1 cells cultured in the DUGL or AGL for 2 days were harvested for transmission electron microscopy (TEM) analysis. The cells were fixed with 2.5% glutaraldehyde at both the locations. Similar to V79 cells (4), the cell samples from both the laboratories were washed with pre-chilled phosphate buffered saline and fixed using 1% OsO_4_. The cells were then dehydrated in ethanol and embedded in epoxy resin. Ultra-thin sections of 80 nm were cut and stained using 3% uranyl acetate and lead citrate. A Hitachi H7650 TEM (Hitachi, Japan) was used to observe the sections.

### Quantification of TMT Protein

The FD-LSC-1 cells were lysed using a lysis buffer [7 M urea, 4% sodium dodecyl sulfate, 30 mM 2-[4-(2-hydroxyethyl) piperazin-1-yl]ethanesulfonic acid, 1 mM phenylmethanesulfonyl fluoride, 2 mM ethylenediaminetetraacetic acid, 10 mM dithiothreitol, and 1 × protease inhibitor cocktail (Sigma-Aldrich, USA)]. The protein lysates were sonicated on ice (5 s pulse on, 15 s pulse off, 180 W power, 10 min). After centrifugation at 10,000 × *g* and 4°C for 10 min, the protein concentrations in the supernatants were determined using a bicinchoninic acid protein assay kit (Fisher Scientific, USA). Hundred microgram of protein from each sample was then reduced using 10 mM dithiothreitol and alkylated using 35 mM iodoacetamide under dark conditions for 30 min. The samples were incubated in pre-chilled (at −20°C) acetone for 3 h and centrifuged at 20,000 × *g* and 4°C for 30 min. The samples were then washed twice with a 50% acetone and 50% ethanol mix and centrifuged at 20,000 × *g* and 4°C for 30 min. The precipitates were resuspended in 100 μL of 100 mM tetraethylammonium bromide and digested twice with trypsin (Promega, USA) at 37°C. The enzyme-protein ratio was 1:100 (w/w).

The digested samples were labeled with 6-plex TMT tag (Thermo Scientific, USA) for 2 h at room temperature, equilibrated to room temperature, and dissolved in 41 μL of anhydrous acetonitrile. The samples from the DUGL groups were labeled with TMT-126, −127, and −128. The samples from the AGL group were labeled with TMT-129, −130, and −131. The samples were then combined and lyophilized. Subsequently, the samples were desalted in a Sep-Pak C18 column (100 mg, 1 cc, Waters, USA) (4).

The labeled samples were fractionated by high performance liquid chromatography in a BEH C18 column (2.1 × 150 mm, 1.7 μm, 130 Å) (Acquity UPLCBEH C18, Waters Corporation, Eschborn, Germany) and a two-mobile-phase gradient elution system (mobile phase A: 10 mM ammoniumformate, pH 10; mobile phase B: 10 mM ammoniumformate and 90% acetonitrile, pH 10). The eluted fractions were collected using an automated fraction collector and combined into twelve fractions.

Peptide analyses were performed using an Orbitrap Fusion mass spectrometer (Thermo Scientific, San Jose, CA, USA) in positive ionization mode using the data-dependent acquisition (DDA) strategy. The full spectrum MS mode was used with the following settings: ESI voltage, 2 kV; capillary temperature, 300°C; automatic gain control target, 5 × 10^5^ ions at a resolution of 70,000; scan range, 350–1,600 m/z; maximum injection time, 50 ms. The MS/MS data were acquired using the top 15 intense parent ions. The resolving power was set to 17,500 at an m/z value of 200. The MS/MS minimum ionic strength was 50,000, and the maximum injection time was 150 ms. The automatic gain control target for MS/MS was 2 × 10^5^ with a 2 Da isolation window.

### Protein Identification and Quantification

Raw data were initially processed using Proteome Discoverer (version 1.4.0.288, Thermo Fisher Scientific, USA), and protein identification was performed using MASCOT (Version 2.3.2, Matrix Science). MASCOT was configured to scan the UniProt database (taxonomy: *Homo sapiens*, total 161,584 entries) assuming digestion by trypsin. The protein identification parameters were the same as those in our previous study: Carbamidomethyl (C), TMT 6-plex (K), and (N-term) were defined as fixed modifications in MASCOT. Oxidation (M) was defined as a variable modification.

Relative quantification of the identified proteins was performed using the weighted ratios of the uniquely identified peptides belonging to a specific protein. The parameters for protein identification and quantification were the same as those in a previous report (4). Fisher's test was used for statistical analysis with a false discovery rate ≤ 1%. Proteins with a *p* < 0.05 and an absolute fold change ≥ 1.2 were considered differentially abundant.

### PRM

The DAPs of TMT with high abundance and fold change were selected for verification. The verification was conducted using a Triple TOF 6,600+ LC-MS/MS system. Protein extraction, lysis, and desalting were performed as described above. The raw DDA files were analyzed using MaxQuant (version 1.3.0.5). The search for the resulting data was performed using the UniProt-cricetulus+griseus fasta database Protein Pilot. The PRM validation data were analyzed using Skyline. The peak shapes of the target peptides were identified by manual inspection.

### Biological Function

The DAPs were functionally annotated using the OmicsBean software (http://www.omicsbean.com:88/). Fisher's exact test was used for the statistical analyses of Gene Ontology (GO) terms and Kyoto Encyclopedia of Genes and Genomes (KEGG) pathways. A corrected *p* < 0.05 was considered to represent significant enrichment. With a confidence cutoff of 400, protein-protein interaction network analyses were performed to further reveal the functional interactions between the DAPs.

### Statistical Analysis

The differences between the OD values of cultures from the DUGL and AGL groups were compared using Student's *t*-test. A *p* < 0.05 was considered statistically significant.

## Results

### Cell Growth and Morphology

**Figure 2:**
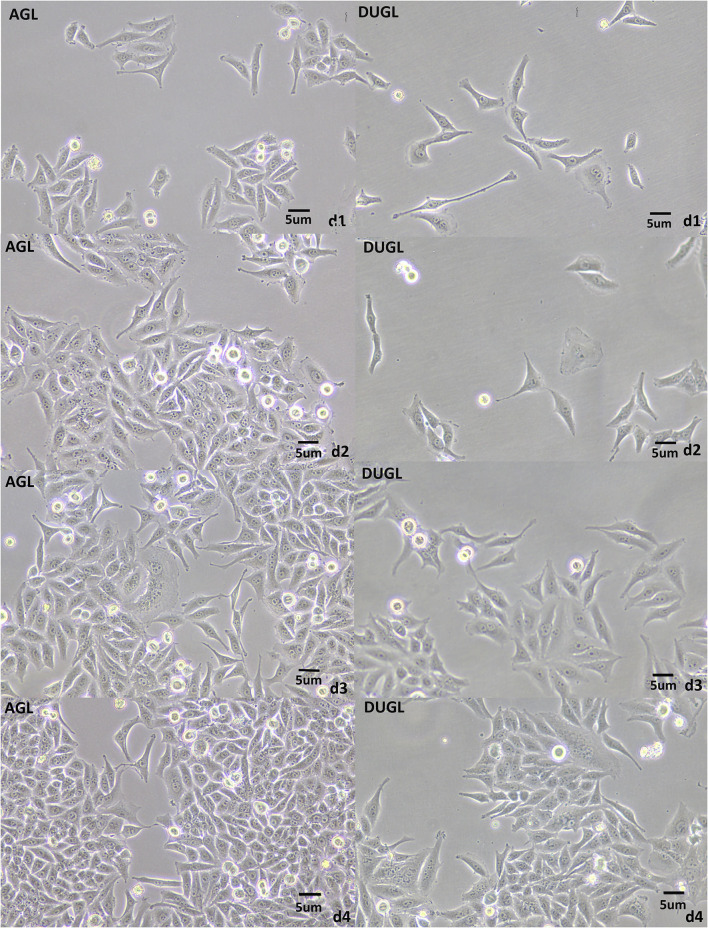
FD-LSC-1 cells cultured for 4 days in the DUGL or AGL observed using light microscopy (at 10 × magnification). AGL, above-ground laboratory; DUGL, deep underground laboratory. The cell density in the DUGL group was observably lower than that in the AGL group on the corresponding day.

**Figure 3:**
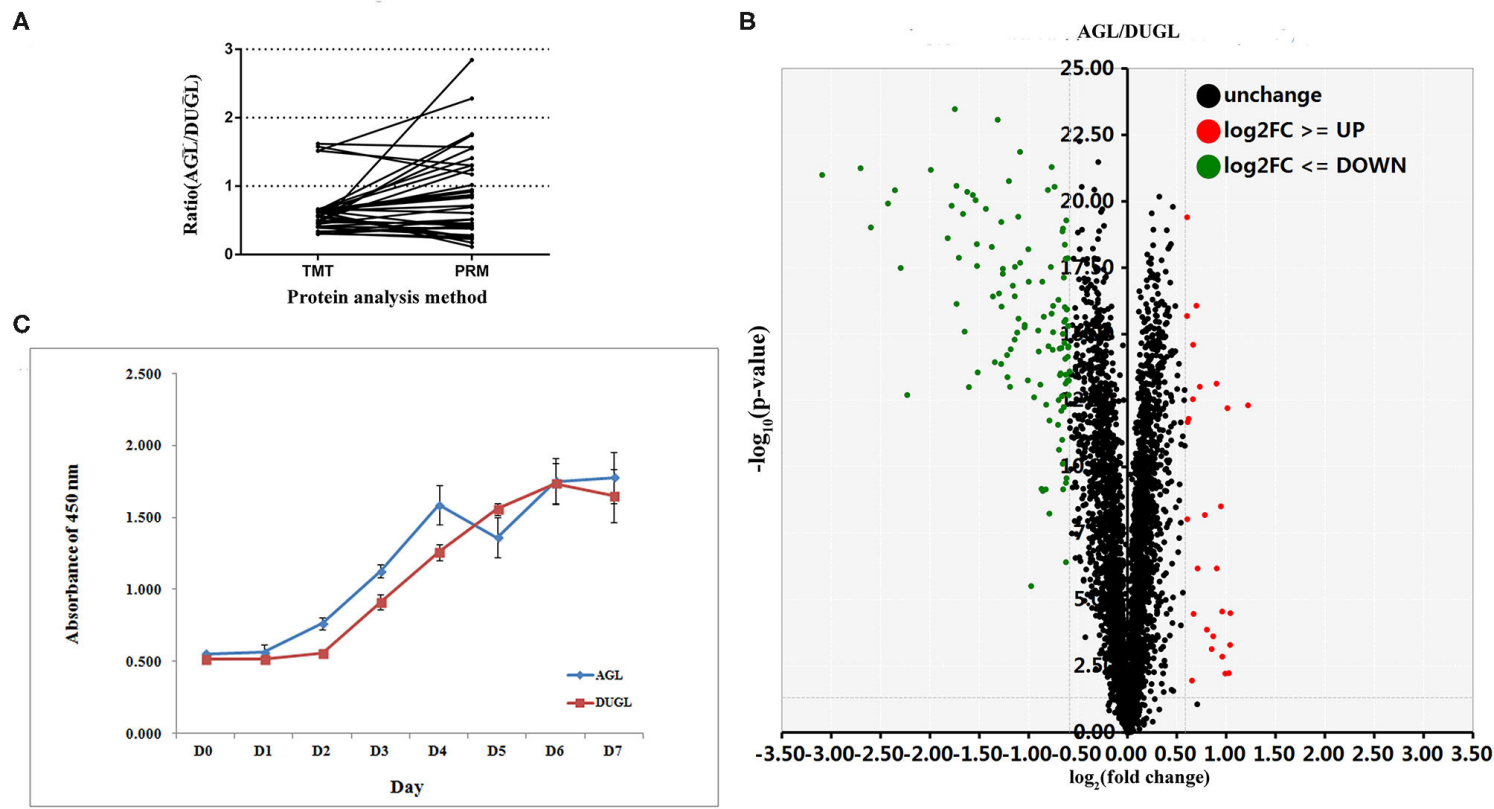
**(A)** PRM verification results of the selected DAPs (AGL/DUGL); **(B)** Growth curves of FD-LSC-1 cells cultured in the DUGL or AGL; **(C)** Volcano plot (red, upregulated DAPs; black, unchanged DAPs; green, downregulated DAPs [AGL/DUGL]). DAPs, differentially abundant proteins; DUGL, deep underground laboratory; AGL, above-ground laboratory; PRM, parallel reaction monitoring.

The proliferation potential of FD-LSC-1 cells cultured in the DUGL was significantly inhibited compared to that of cells cultured in the AGL. After 2 days of culture, the OD_450_ value of FD-LSC-1 cells cultured in the AGL (OD value: AGL/DUGL = 0.763 ± 0.045/0.557 ± 0.049, *p* < 0.001) increased by 37.73%, whereas that of cells cultured in the DUGL increased only by 7.53%. On the third day, the OD value of FD-LSC-1 cells cultured in the AGL doubled, whereas that of cells cultured in the DUGL only increased by 76.25% (AGL/DUGL = 1.126 ± 0.136/0.913 ± 0.056, *p* < 0.001). The FD-LSC-1 cultures from the AGL reached maximum saturation density (AGL/DUGL = 1.586 ± 0.13/1.26 ± 0.042, *p* < 0.001) in the 96-well plate on the fourth day, whereas the cultures from the DUGL attained the same (AGL/DUGL = 1.360 ± 0.159/1.558 ± 0.140) on the fifth day (Figures 2, 3C).

**Figure 4:**
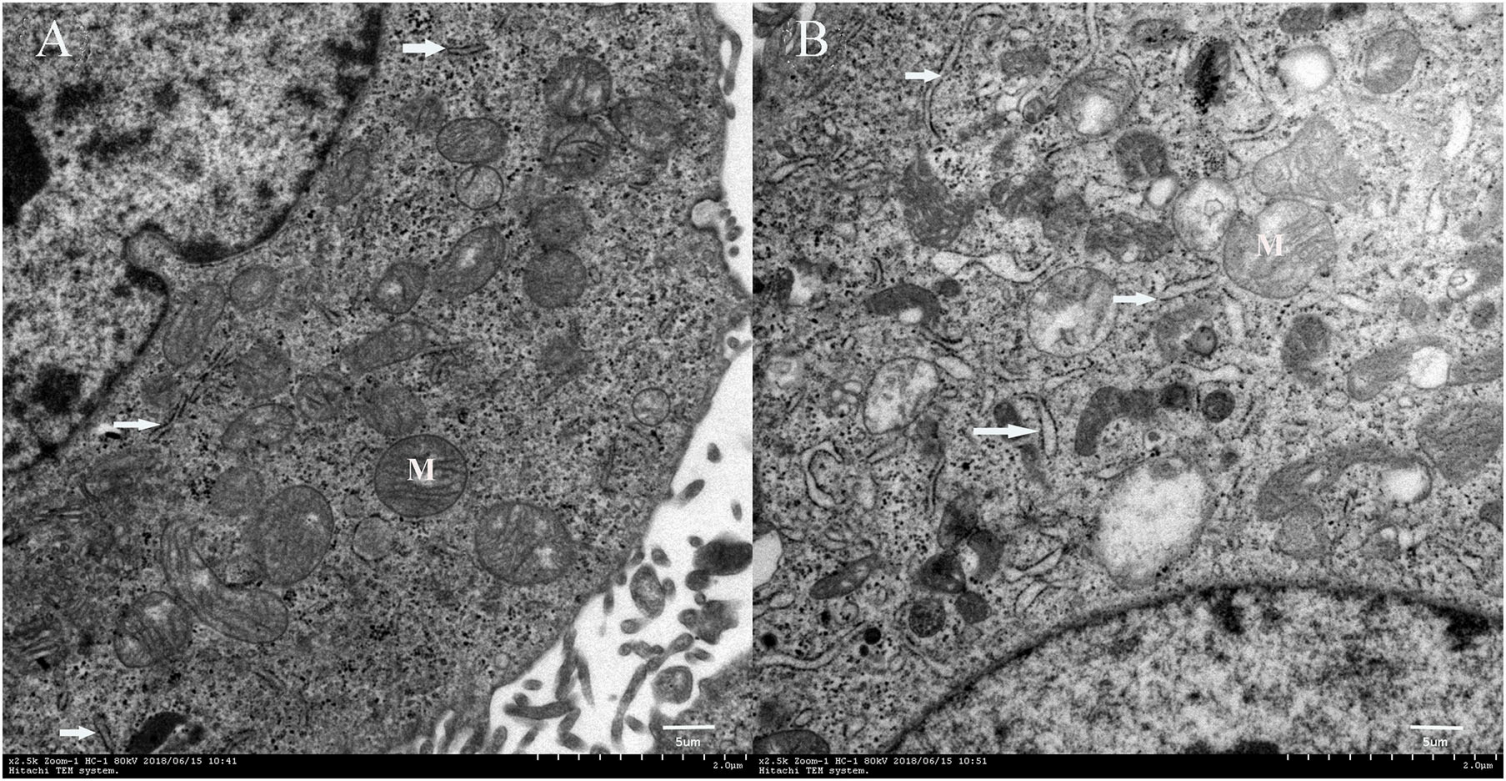
Transmission electron microscopy images of FD-LSC-1 cells cultured in the AGL **(A)** and DUGL **(B)** (at 2,500 × magnification). White arrows, endoplasmic reticulum; M, mitochondria; AGL, above-ground laboratory; DUGL, deep underground laboratory.

The TEM scans showed that cells cultured in the DUGL contained hypertrophic endoplasmic reticulum (ER) as well as a higher number of ER compared to the cells cultured in the AGL (Figure 4).

### Quantitative Proteomic Analyses

In the quantitative proteomic analyses, 37,202 unique spectra were matched to 29,719 unique peptides. In addition, 4,094 unique proteins were identified in FD-LSC-1 cells cultured in the DUGL and AGL. Eight hundred and seven DAPs were detected with a relative abundance absolute fold change cutoff ≥ 1.2 and *p* < 0.05. Of these, 536 proteins were upregulated (from 1.2 to 8.5-fold) and 271 proteins were downregulated (from 0.83 to 0.43-fold) in the FD-LSC-1 cells cultured in the DUGL compared to those cultured in the AGL (Figure 3B, Supplementary Table 1). One hundred and forty-five DAPs were identified with a absolute fold change cutoff ≥ 1.5 and *p* < 0.05 (118 upregulated and 27 downregulated in cells cultured in the DUGL). The protein names, accession numbers, and abbreviations used in our proteomic analyses were obtained from the UniProtKB/Swiss-Prot database. Proteomic data were deposited in the ProteomeXchange Consortium (Subproject: IPX0002120001).

### Functional Analysis of DAPs

GO term enrichment and KEGG pathway analyses were performed to investigate the biological functions of the DAPs in FD-LSC-1 cells from the DUGL and AGL groups.

**Figure 5:**
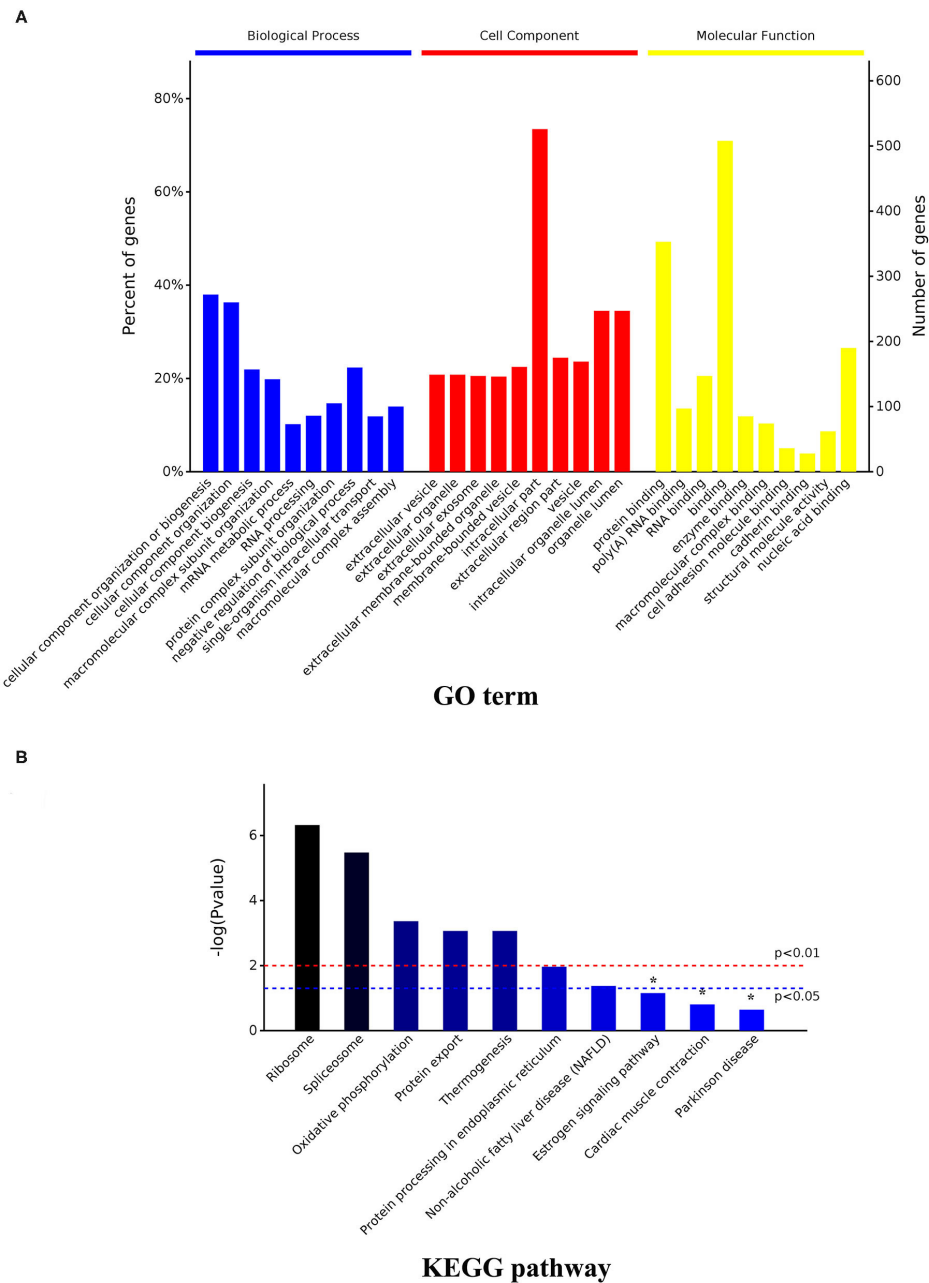
GO and KEGG enrichment analysis of the total DAPs. **(A)** GO analysis result; **(B)** KEGG pathway analysis result. DAPs, differentially abundant proteins.

**Figure 6:**
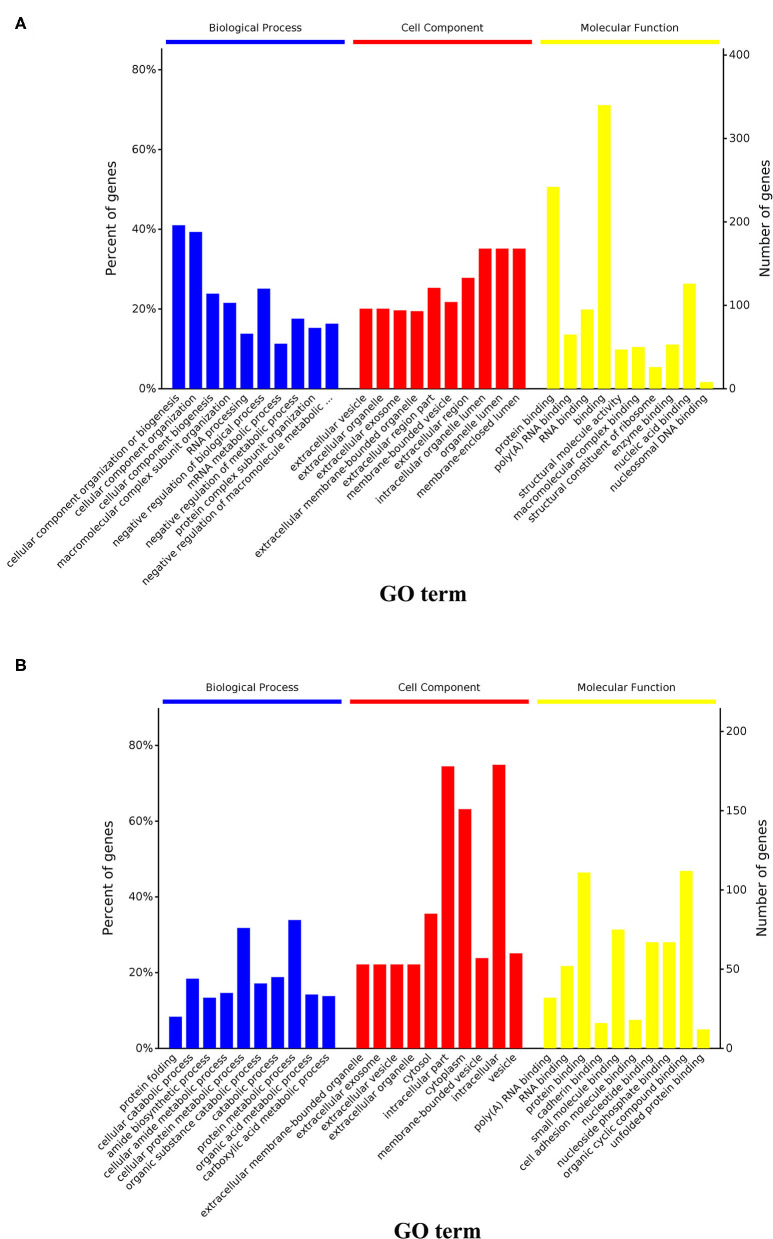
GO and KEGG enrichment analysis of the upregulated and downregulated DAPs. **(A)** Results of GO analysis of upregulated DAPs in cells from the DUGL group; **(B)** Results of GO analysis of downregulated DAPs in cells from the DUGL group. DAPs, differentially abundant proteins.

At a false discovery rate (*p*-adjusted) < 0.05, the GO term enrichment analysis of DAPs revealed that the top five enriched GO terms were cellular component organization or biogenesis, cellular component organization, cellular component biogenesis, macromolecular complex subunit organization, and mRNA metabolic process in the biological process (BP) category; extracellular vesicle, organelle, exosome, membrane-bounded organelle, membrane-bounded vesicle in the cellular components (CC) category; and protein binding, poly(A) RNA binding, RNA binding, binding, and enzyme binding in the molecular functions (MF) category (Figure 5A, Supplementary Table 2). The GO enrichment analysis of upregulated DAPs in cells cultured in the DUGL showed that the top five GO terms from the BP category were cellular component organization or biogenesis, cellular component organization, cellular component biogenesis, macromolecular complex subunit organization, and RNA processing. The terms in the CC category were extracellular vesicle, organelle, exosome, membrane-bounded organelle, and region part (Figure 6A, Supplementary Table 3). The terms in the MF category were protein binding, poly(A) RNA binding, RNA binding, binding, and structural molecule activity. In contrast, the top five GO terms for downregulated DAPs in cells cultured in the DUGL were protein folding, cellular catabolic process, amide biosynthetic process, cellular amide metabolic process, and cellular protein metabolic process in the BP category (Figure 6B, Supplementary Table 4); extracellular membrane-bounded organelles, exosomes, vesicles, organelles, and cytosol in the CC category; and poly(A) RNA binding, RNA binding, protein binding, cadherin binding, and small molecule binding in the MF category.

With a *p*-adjusted value < 0.05, the KEGG pathway analysis of these DAPs revealed that seven pathways were enriched. The significantly enriched pathways were ribosome (*p* < 0.0001), spliceosome (*p* = 0.0001), oxidative phosphorylation (OXPPL) (*p* = 0.0001), protein export (*p* = 0.0001), thermogenesis (*p* = 0.0003), protein processing in the endoplasmic reticulum (*p* = 0.0108), and non-alcoholic fatty liver disease (*p* = 0.0421) (Figure 5B, Table 1).

**Table 1:** KEGG pathway enrichment result.

**Pathway**	***p*-value (adjusted)**	**Number of upregulated proteins (n)**	**Upregulated DAPs**	**Number of downregulated proteins (n)**	**Downregulated DAPs**
Ribosome	*p <* 0.0001	17	RPL39, RPS28, RPL30, RPL37A, RPL22, RPL38, RPL35A, RPS12, RPL34, RPS25, RPS27, RPL36, RPL26, MRPS17, RPS26, RPLP2, RPL36A	3	RPL7, RPS2, RPL4
Spliceosome	0.0001	13	SF3A2, SNRPF, DDX46, SLU7, ZMAT2, TRA2A, SMNDC1, LSM6, LSM5, SRSF3, SRSF5, SNRPD2, LSM2	5	HSPA8, PRPF40A, ISY1, PCBP1, HNRNPA1
Oxidative phosphorylation	0.00043	14	COX6C, UQCR10, ATP5ME, NDUFB3, NDUFAB1, NDUFS5, UQCRH, NDUFB1, ATP5PF, ATP5F1E, COX4I1, NDUFA2, COX6B1, ATP6V1G1	0	
Protein export	0.00086	2	SEC61B, SRP19	4	SRPRA, SRPRB, HSPA5, SRP72
Thermogenesis	0.00086	17	COX6C, UQCR10, COX19, COA7, ATP5ME, KDM3B, SMARCD2, NDUFB3, COX20, NDUFAB1, NDUFS5, UQCRH, NDUFB1, ATP5PF, ATP5F1E, NDUFA2, COX6B1	1	PRKACA
Protein processing in endoplasmic reticulum	0.00108	3	RBX1, SEC61B, WFS	10	EDEM3, HSPA8, ERP29, DNAJA1, P4HB, CANX, HSPA5, STT3B, HSP90AA1, HSP90AB1
Non-alcoholic fatty liver disease	0.0421	11	COX6C, UQCR, NDUFB, NDUFAB1, BID, NDUFS5, UQCRH, NDUFB1, COX4I1, NDUFA, COX6B1	0	

### PRM Verification of DAPs

Out of the DAPs identified by PRM, 77.14% (27/35) were consistent with those identified in the TMT proteomic analysis. This indicated that the TMT proteomic analyses yielded reliable results (Figure 3A, Table 2).

**Table 2:** Results of DAP verification by parallel reaction monitoring.

**UniProt ID**	**Gene name**	**TMT (AGL/DUGL)**	**PRM (AGL/DUGL)**
A0A087WW43	ITIH3	0.4017319	2.844
A0A0G2JPA8	SERPINF2	0.3238109	0.512
A8MZH8	PTTG1IP	0.665582	0.86
E9PES6	HMGB3	0.6464124	0.944
E9PIT3	F2	0.6469235	0.383
E9PS74	SLC43A3	1.519244	1.302
F8W696	APOA1	0.3153911	0.227
I3L4G8	CHMP6	0.6435466	1.76
O00622	CYR61	0.4987531	0.427
P01023	A2M	0.411034	0.514
P01024	C3	0.390117	0.282
P02461	COL3A1	0.4081448	0.378
P05543	SERPINA7	0.3045479	0.259
P09669	COX6C	0.6596789	0.87
P09923	ALPI	1.57784	1.171
P13645	KRT10	0.4975399	1.243
P13647	KRT5	0.6197494	0.174
P14324	FDPS	1.619579	1.568
P17096	HMGA1	0.6389323	0.714
P35527	KRT9	0.4700966	0.457
P35908	KRT2	0.5715737	0.253
P43652	AFM	0.2987056	0.418
P60903	S100A10	0.5489479	0.246
P61769	B2M	0.633045	0.113
P81605	DCD	0.6339814	0.917
P98179	RBM3	0.4456107	0.694
Q01469	FABP5	0.6432707	1.304
Q15428	SF3A2	0.6603566	1.41
Q4KMP7	TBC1D10B	0.4632966	1.747
Q5T985	ITIH2	0.3368894	0.393
Q6ZVX7	NCCRP1	1.517451	2.281
Q8WXA9	SREK1	0.5544262	1.554
Q96B23	C18orf25	0.6465517	0.837
Q9BXV9	GON7	0.6654836	0.607
Q9UPY3	DICER1	0.574236	1.017

### Protein-Protein Interaction Network Construction and Module Analysis

**Figure 7:**
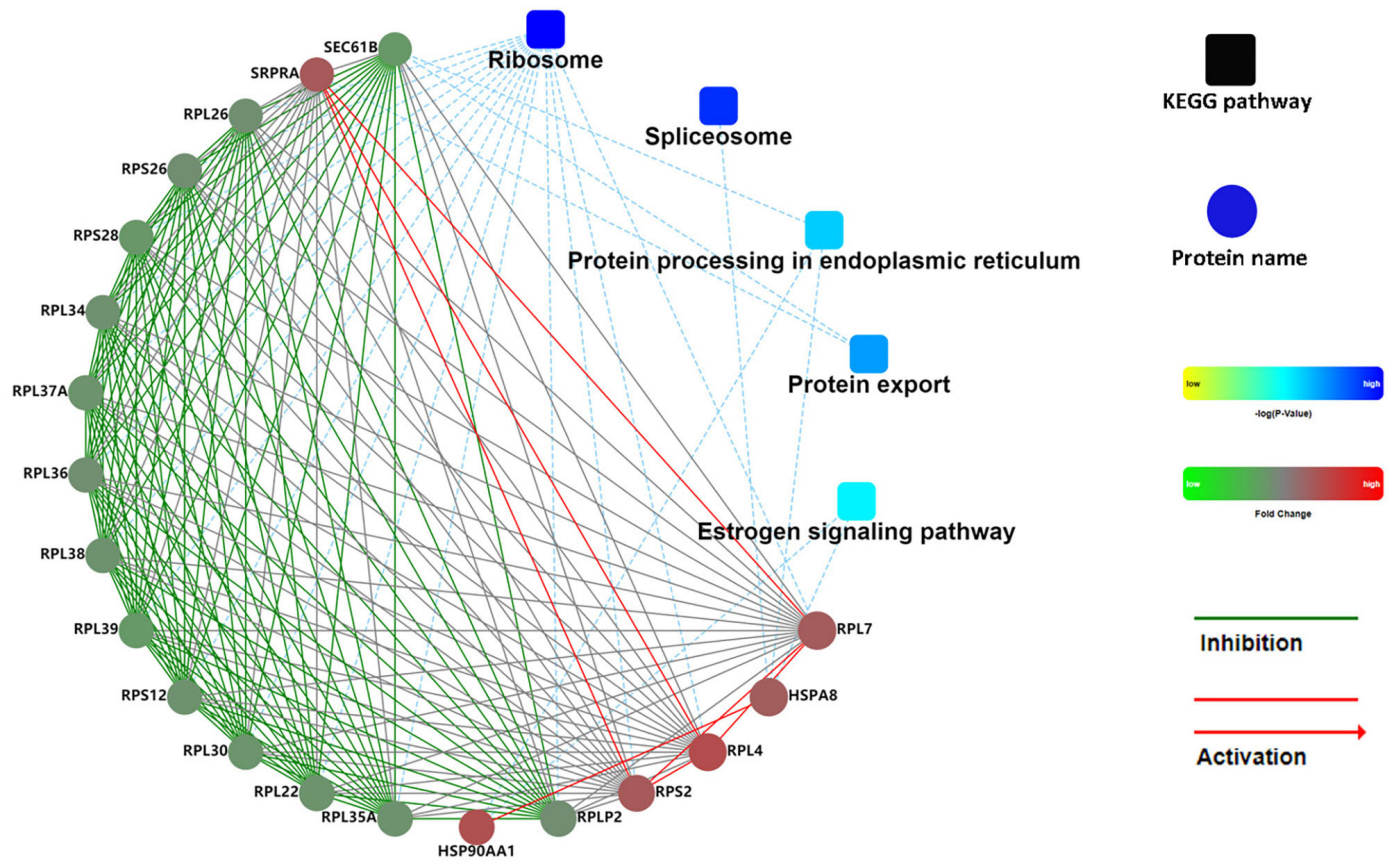
PPI network of DAPs in FD-LSC-1 cells. Coloration with gradient: from yellow (lower *p*-value) to blue (higher *p*-value). The proteins with different abundances are indicated in red (upregulation in the AGL group) and green (downregulation in the AGL group). The default confidence cutoff value was fixed at 400. The red solid lines represent activation. The blue dashed lines indicate the KEGG pathway. PPI, protein-protein interaction; DAPs, differentially abundant proteins.

To further ascertain the functional interactions between the DAPs, the Cytoscape software was used to construct the protein-protein interaction networks with a confidence cutoff of 400 (Figure 7). The findings showed that ribosome-related proteins were highly interrelated and played central roles across the network.

## Discussion

To our knowledge, this is the first study to investigate the biological effects of cancer cells cultured in a deep underground environment, and provide novel insights into cancer. Although relative humidity, air pressure, and concentration of CO_2_ and radon gas were significantly different between DUGL and AGL, the cell culture incubators at both the locations had the same level of relative humidity, temperature, and concentration of CO_2_ (4). The air pressure (1.2–6 bar) could enhance cell growth rate (5, 6). Air pressure in the DUGL (1118.2 hPa) was slightly higher than that in the AGL (951.9 hPa); however, the difference was dramatically reduced by the shift in gas and liquid (4). After all, the cells were cultured in liquid medium in the incubators. The 1,400-m rocky cover over of the DUGL shields almost all the cosmic radiation (7). Moreover, the terrestrial radiation in DUGL (0.04 μSv/h) was significantly lower than that in AGL (0.15 μSv/h) (4). Radon concentration (1.5 pCi/L) of the DUGL was similar to that at Gran Sasso National Laboratory (LNGS) and less than the normal background (1.7 nGy/h:0.04 μSv/h) (4). Herein, we speculate that BBR, as the main environmental factor, affected the cultures in the DUGL at the CJEM.

Consistent with previous reports, the present study confirmed that BBR exposure in a deep underground environment can reduce the growth rates of cells, even cancer cells, within a short duration (8–11). Additionally, we identified 807 DAPs (536 proteins upregulated and 271 proteins downregulated in FD-LSC-1 cells cultured in the DUGL) with a 1.2 absolute fold change, and 145 DAPs (118 upregulated and 27 downregulated in cells cultured in the DUGL) with a 1.5 absolute fold change. The upregulated DAPs underwent fold change ranging from 1.2- to 8.5-fold, while the downregulated DAPs underwent fold change ranging from 0.83- to 0.43-fold. Meanwhile, the ER was observed to be hypertrophic and present at a greater number in FD-LSC-1 cells cultured in the DUGL as observed using TEM. The changes in the proteomics profile and morphology of ER were also similar to those of V79 cells cultured in the DUGL (4), which strongly suggested that BBR could promote protein synthesis in cultures within a short duration.

The GO enrichment analyses helped us elucidate the functions of the DAPs (12). In the BP category, cellular component organization or biogenesis, macromolecular complex subunit organization, and mRNA metabolic process were the top three enriched terms. In addition, the upregulated DAPs were significantly enriched for negative regulation of biological and metabolic processes, which might explain the increased protein expression and delayed growth of FD-LSC-1 cells cultured in the DUGL. In the MF category, the poly(A) RNA binding, RNA binding, protein binding, and binding terms were enriched significantly, consistent with our observations in V79 cells used in our previous research. This suggested that BBR exposure in a deep underground environment primarily affected the macromolecular functions of the cultures by initiating binding dysregulation, especially in RNAs and proteins. The FD-LSC-1 cells cultured in DUGL exhibited enrichment in extracellular terms (e.g., vesicle, organelle, exosome, and membrane-bounded organelle) in the CC category, contrary to the V79 cells, which exhibited enrichment in intracellular terms. This implied that different cells exhibited similar or different responses at the molecular level under BBR exposure in a deep underground environment.

KEGG pathway analyses have been widely used to systematically identify the functions of large-scale proteins and/or genes in cells. Consistent with the observations in V79 cells in our previous research (4), the KEGG analyses of FD-LSC-1 DAPs showed that ribosome and spliceosome were the two most significantly enriched pathways. Meanwhile, most DAPs enriched in the two pathways were upregulated in both V79 and FD-LSC-1 cells cultured in the DUGL. Extracellular stimulation can induce ribosomal stress, disturb ribosome biogenesis, and affect cell proliferation (13). Similar to that in V79 cells, both RPL26, and RPS27, which regulate p53 expression and cell cycle arrest, were upregulated in cells cultured in the DUGL groups. These findings further indicate that ribosomal proteins, especially RPL26, and RPS27, play a significant role in the cellular stress response induced upon exposure to BBR in a deep underground environment and inhibit the proliferative potential of cells through p53 (14).

Spliceosome is an essential cellular organelle for cell growth and division that regulates gene expression and protein synthesis (15). The catalysis of precursor mRNA splicing by spliceosomes, a multi-megadalton ribonucleoprotein complex (16), is a vital step in the flow of genetic information from DNA to proteins in eukaryotes (17). We observed that 15/28 DAPs enriched in the spliceosome pathway were upregulated in cells cultured in the DUGL. Among the upregulated DAPs, seven proteins (ZMAT2, PRPF40A, SNRPD2, SLU7, SRSF5, SRSF3, and SNRPF) followed the same expression model as that in V79 cells (4). Pre-mRNA processing factor 40 homolog A (PRPF40A) plays a crucial role in the initiation of pre-mRNA splicing (18), while the splicing regulator SLU7 serves as an essential factor for the maintenance of cell viability (19). These findings further indicate that BBR could induce protein expression by enhancing spliceosome function, which might be advantageous for cell survival in an altered environment.

The ER has multiple functions, including the synthesis, folding, quality control, and transport of one-third of all cellular proteins (20, 21). In addition, the ER is a critical site for calcium homeostasis and can respond to exogenous stimuli by perceiving environmental signals (22). In the present study, 10/13 proteins enriched in protein processing in the ER pathway were downregulated in cells cultured in the DUGL, which was consistent with the findings from our previous analyses conducted on V79 cells (4). Notably, five proteins (WFS1, STT3B, CANX, ERP29, and HSPA5) were downregulated both in V79 and FD-LSC-1 cells cultured in the DUGL. Wolfram syndrome 1 gene (WFS1), an ER-resident transmembrane protein, regulates the cellular response to ER stress (23). The knockdown of WFS1 can render cells vulnerable to oxidative stress and induce alterations in mitochondrial homeostasis (24). Post-translational N-glycosylation mediated by STT3B plays an important role in the quality control of non-glycosylated proteins. The knockdown of STT3B was observed to delay cell proliferation (25). As an integral chaperon protein of the ER, calnexin (CANX) promotes cancer cell growth and proliferation. Endoplasmic reticulum protein 29 (ERp29), which plays a role in protein unfolding and secretion, is associated with the ER stress response (26). Heat shock protein 5 (HSPA5/GRP78/BiP) protects cells against ER stress and damage caused by reactive oxygen species, and helps cells survive by regulating calcium signaling in mitochondria-associated ER membranes (27). These findings strongly indicate that the ER plays a central role in the BBR stress response, and the downregulation of major ER stress proteins might explain the reduction in stress tolerance potential observed in previous studies (28). Additionally, the downregulation of proliferation-promoting proteins might further contribute to the delay in the growth of cells cultured under low background radiation in the DUGL.

The activation of ribosomes and spliceosomes requires energy (29). Mitochondria can supply energy for cellular survival and development. Mitochondrial OXPPL is the primary pathway for energy synthesis in all cell types (30). In the current study, we characterized 14 DAPs that were upregulated in FD-LSC-1 cells cultured in the DUGL. The DAPs that underwent maximum upregulation were associated with energy metabolism related to thermogenesis and non-alcoholic fatty liver disease pathways. These results suggested that under BBR stress, FD-LSC-1 cells could activate the OXPPL pathway and enhance energy supply for cell survival. In support of this proposition, we observed that the expression of ubiquinol cytochrome c reductase hinge (UQCRH) and ATP6V1G1 also increased in V79 cells cultured under BBR (4). UQCRH upregulation suggests an increase in mitochondrial OXPPL activity (31). However, the findings of this study are contrasting to those of a previous study showing that some of the proteins enriched in the OXPPL pathway were downregulated in V79 cells and *Shewanella oneidensis* along with downregulation of ATPase mRNA (32). Cytochrome oxidase subunit 6B1 (COX6B1), which protects cells against injury (33, 34), was downregulated in V79 cells and upregulated in FD-LSC-1 cells cultured under BBR. The inconsistent results indicate that different cells express the same proteins as well as different proteins at different time points to counter BBR stress. UQCRH and ATP6V1G1 might be critical for cellular stress response under BBR. Notably, Carbone et al. (1) reported the inhibition in the activity of superoxide dismutase in TK6 cells cultured in the BBR environment in LNGS. This indicated the inhibition of superoxide dismutase expression, as observed through proteomic analyses in our study.

## Conclusion

BBR exposure in a deep underground environment could inhibit the proliferation of FD-LSC-1 cells. In response, the FD-LSC-1 cells presented with changes in the proteomic profile related to the ribosome, gene spliceosome, RNA transport, and energy metabolism among others. The alterations in protein expression might constitute the molecular basis of proliferation inhibition and enhanced survivability in cells adapting to BBR in a deep underground environment. RPL26, RPS27, ZMAT2, PRPF40A, SNRPD2, SLU7, SRSF5, SRSF3, SNRPF, WFS1, STT3B, CANX, ERP29, HSPA5, COX6B1, UQCRH, and ATP6V1G1 were the major proteins associated with the BBR stress response in cells. Our findings provide novel insights into the response of cancer cells from a different perspective, in which the usual levels of radiation were reduced drastically in a deep underground environment.

## Limitations

Similar to our previous reports, our present study has certain limitations. First, the growth curve obtained after culturing cells in the deep underground environment represented data recorded only for a week, and the samples used for the proteomic analyses were cultured for only 4 days in the DUGL. Second, the functions of DAPs should be verified in knockdown and overexpression studies. Third, it is necessary to use a normal cell line as a control in future studies. Fourth, some of the environmental factors could not be regulated to the desired levels owing to the challenge of ventilation in deep mines.

## Data Availability Statement

The datasets presented in this study can be found in online repositories. The names of the repository/repositories and accession number(s) can be found in the article/Supplementary Material.

## Author Contributions

JiL, HX, SL, JZ, MG, JW, and JuL conceived and designed the project. TM, SW, YX, JC, LW, YL, and QW performed all the experiments. LJ wrote the manuscript and prepared the figures. JW, MG, WL, and HX reviewed and revised the manuscript. All authors reviewed and approved the manuscript.

## Conflict of Interest

The authors declare that the research was conducted in the absence of any commercial or financial relationships that could be construed as a potential conflict of interest.

## References

[1] Carbone MC Pinto M Antonelli F Amicarelli F Balata M Belli M. The cosmic silence experiment: on the putative adaptive role of environmental ionizing radiation. Radiat Environ Biophys. (2009) 48:189–96. 10.1007/s00411-008-0208-6 19169701

[2] Huang L Kim PM Nickoloff JA Morgan WF. Targeted and nontargeted effects of low-dose ionizing radiation on delayed genomic instability in human cells. Cancer Res. (2007) 67:1099–104. 10.1158/0008-5472.CAN-06-3697 17283143

[3] Liu J Ma T Liu Y Zou J Gao M Zhang R. History, advancements, and perspective of biological research in deep-underground laboratories: a brief review. Environ Int. (2018) 120:207–14. 10.1016/j.envint.2018.07.031 30098554

[4] Liu J Ma T Gao M Liu Y Liu J Wang S. Proteomics provides insights into the inhibition of Chinese hamster V79 cell proliferation in the deep underground environment. Sci Rep. (2020) 10:14921. 10.1038/s41598-020-71154-z 32913333 PMC7483447

[5] Pinheiro R Belo I Mota M. Growth and beta-galactosidase activity in cultures of Kluyveromyces marxianus under increased air pressure. Lett Appl Microbiol. (2003) 37:438–42. 10.1046/j.1472-765X.2003.01429.x 14633095

[6] Pinheiro R Belo II Mota M Air pressure effects on biomass yield of two different Kluyveromyces strains. Enzyme Microb Technol. (2000) 26:756–62. 10.1016/S0141-0229(00)00168-X 10862882

[7] Thome C Tharmalingam S Pirkkanen J Zarnke A Laframboise T Boreham DR. The REPAIR project: examining the biological impacts of sub-background radiation exposure within SNOLAB, a deep underground laboratory. Radiat Res. (2017) 188:470–4. 10.1667/RR14654.1 28723273

[8] Planel H Soleilhavoup JP Tixador R Richoilley G Conter A Croute F Influence on cell proliferation of background radiation or exposure to very low, chronic gamma radiation. Health Phys. (1987) 52:571–8. 10.1097/00004032-198705000-00007 3106264

[9] Smith GB Grof Y Navarrette A Guilmette RA. Exploring biological effects of low level radiation from the other side of background. Health Phys. (2011) 100:263–5. 10.1097/HP.0b013e318208cd44 21595063

[10] Castillo H Schoderbek D Dulal S Escobar G Wood J Nelson R. Stress induction in the bacteria Shewanella oneidensis and deinococcus radioduransin response to below-background ionizing radiation. Int J Radiat Biol. (2015) 91:749–56. 10.3109/09553002.2015.1062571 26073528

[11] Kawanishi M Okuyama K Shiraishi K Matsuda Y Taniguchi R Shiomi N Growth retardation of Paramecium and mouse cells by shielding them from background radiation. J Radiat Res. (2012) 53:404–10. 10.1269/jrr.11145 22739010

[12] Camon E Magrane M Barrell D Lee V Dimmer E Maslen J. The gene ontology annotation (GOA) database: sharing knowledge in uniprot with gene ontology. Nucleic Acids Res. (2004) 32:D262–6. 10.1093/nar/gkh021 14681408 PMC308756

[13] Zhou X Liao WJ Liao JM Liao P Lu H. Ribosomal proteins: functions beyond the ribosome. J Mol Cell Biol. (2015) 7:92–104. 10.1093/jmcb/mjv014 25735597 PMC4481666

[14] Ma XR Sim UH Ling TY Tiong TS Khoo SB. Expression trend of selected ribosomal protein genes in nasopharyngeal Carcinoma. Malays J Med Sci. (2012) 18:23–30. 23613646 PMC3629677

[15] Li F Zhao D Yang S Wang J Liu Q Jin X. ITRAQ-based proteomics analysis of triptolide on human A549 lung adenocarcinoma cells. Cell Physiol Biochem. (2018) 45:917–34. 10.1159/000487286 29428961

[16] Will CL Lührmann R Spliceosome structure and function. Cold Spring Harbor Perspect Biol. (2011) 3:a003707 10.1101/cshperspect.a003707 PMC3119917 21441581

[17] Shi Y Mechanistic insights into precursor messenger RNA splicing by the spliceosome. Nat Rev Mol Cell Biol. (2017) 18:655–70. 10.1038/nrm.2017.86 28951565

[18] Huo Z Zhai S Weng Y Qian H Tang X Shi Y. PRPF40A as a potential diagnostic and prognostic marker is upregulated in pancreatic cancer tissues and cell lines: an integrated bioinformatics data analysis. OncoTargets Ther. (2019) 12:5037–51. 10.2147/OTT.S206039 31303762 PMC6610298

[19] Urtasun R Elizalde M Azkona M Latasa MU García-Irigoyen O Uriarte I. Splicing regulator SLU7 preserves survival of hepatocellular carcinoma cells and other solid tumors via oncogenic miR-17-92 cluster expression. Oncogene. (2016) 35:4719–29. 10.1038/onc.2015.517 26804174

[20] Turnham RE Scott JD. Protein kinase A catalytic subunit isoform PRKACA; history, function and physiology. Gene. (2016) 577:101–8. 10.1016/j.gene.2015.11.052 26687711 PMC4713328

[21] Wang Z Feng Y Li J Zou J Fan L. Integrative microRNA and mRNA analysis reveals regulation of ER stress in the Pacific white shrimp litopenaeus vannamei under acute cold stress. Comp Biochem Physiol Part D Genomics Proteomics. (2019) 33:100645. 10.1016/j.cbd.2019.100645 31794884

[22] Ryan D Carberry S Murphy ÁC Lindner AU Fay J Hector S Calnexin, an ER stress-induced protein, is a prognostic marker and potential therapeutic target in colorectal cancer. J Transl Med. (2016) 14:196 10.1186/s12967-016-0984-8 27369741 PMC4930591

[23] Sakakibara Y Sekiya M Fujisaki N Knockdown of wfs1, a fly homolog of Wolfram syndrome 1, in the nervous system increases susceptibility to age- and stress-induced neuronal dysfunction and degeneration in Drosophila. PLoS Genet. (2018) 14:e1007196 10.1371/journal.pgen.1007196 29357349 PMC5794194

[24] Cagalinec M Liiv M. Role of mitochondrial dynamics in neuronal development: mechanism for wolfram syndrome. PLoS Biol. (2016) 14:e1002511. 10.1371/journal.pbio.1002511 27434582 PMC4951053

[25] Sato T Sako Y Sho M Momohara M Suico MA Shuto T STT3B-dependent posttranslational N-glycosylation as a surveillance system for secretory protein. Mol Cell. (2012) 47:99–110. 10.1016/j.molcel.2012.04.015 22607976

[26] Guo L Ma L Liu C Lei Y Tang N Huang Y. ERp29 counteracts the suppression of malignancy mediated by endoplasmic reticulum stress and promotes the metastasis of colorectal cancer. Oncol Rep. (2019) 41:1603–15. 10.3892/or.2018.6943 30569094 PMC6365697

[27] Kim SY Kim HJ Kim HJ Kim DH Han JH Byeon HK. HSPA5 negatively regulates lysosomal activity through ubiquitination of MUL1 in head and neck cancer. Autophagy. (2018) 14:385–403. 10.1080/15548627.2017.1414126 29260979 PMC5915028

[28] Carbone MC Pinto M Antonelli F Balata M Effects of deprivation of background environmental radiation on cultured human cells. Il Nuovo Cimento. (2010) 125:469–77. 10.1393/ncb/i2010-10889-y

[29] Shi Y. The spliceosome: a protein-directed metalloribozyme. J Mol Biol. (2017) 429:2640–53. 10.1016/j.jmb.2017.07.010 28733144

[30] Yan G Li X Cheng X Peng Y Long B Fan Q. Proteomic profiling reveals oxidative phosphorylation pathway is suppressed in longissimus dorsi muscle of weaned piglets fed low-protein diet supplemented with limiting amino acids. Int J Biochem Cell Biol. (2016) 79:288–97. 10.1016/j.biocel.2016.08.024 27590855

[31] Huang YP Chang NW PPARα modulates gene expression profiles of mitochondrial energy metabolism in oral tumorigenesis. BioMedicine. (2016) 6:3 10.7603/s40681-016-0003-7 26869356 PMC4751096

[32] Castillo H Li X Schilkey F Smith GB. Transcriptome analysis reveals a stress response of Shewanella oneidensis deprived of background levels of ionizing radiation. PLoS ONE. (2018) 13:e0196472. 10.1371/journal.pone.0196472 29768440 PMC5955497

[33] Zhang W Wang Y Wan J Zhang P Pei F. COX6B1 relieves hypoxia/reoxygenation injury of neonatal rat cardiomyocytes by regulating mitochondrial function. Biotechnol Lett. (2019) 41:59–68. 10.1007/s10529-018-2614-4 30311029 PMC6313357

[34] Yang S Wu P Xiao J Jiang L. Overexpression of COX6B1 protects against I/R-induced neuronal injury in rat hippocampal neurons. Mol Med Rep. (2019) 19:4852–62. 10.3892/mmr.2019.10144 31059068 PMC6522897

